# scRNA‐seq and proteomics reveal the distinction of M2‐like macrophages between primary and recurrent malignant glioma and its critical role in the recurrence

**DOI:** 10.1111/cns.14269

**Published:** 2023-05-17

**Authors:** Guiting You, Zhenyu Zheng, Yulong Huang, Guifen Liu, Wei Luo, Jianhuang Huang, Longjin Zhuo, Binghua Tang, Shunyi Liu, Caihou Lin

**Affiliations:** ^1^ Department of Neurosurgery Fujian Medical University Union Hospital Fuzhou China; ^2^ Fujian Medical University Fuzhou China; ^3^ Department of Gynaecology, Fujian Provincial Maternity and Children's Hospital Affiliated Hospital of Fujian Medical University Fuzhou China; ^4^ Department of Neurosurgery Affiliated Hospital of Putian University Putian China; ^5^ Pingtan Comprehensive Experimental Area Hospital Fuzhou China

**Keywords:** immune microenvironment, intercellular interaction, recurrent malignant glioma, single‐cell RNA sequencing, tumor‐associated macrophages

## Abstract

**Aims:**

Tumor‐associated macrophages (TAMs) in the immune microenvironment play an important role in the increased drug resistance and recurrence of malignant glioma, but the mechanism remains incompletely inventoried. The focus of this study was to investigate the distinctions of M2‐like TAMs in the immune microenvironment between primary and recurrent malignant glioma and its influence in the recurrence.

**Methods:**

We employed single‐cell RNA sequencing to construct a single‐cell atlas for a total of 23,010 individual cells from 6 patients with primary or recurrent malignant glioma and identified 5 cell types, including TAMs and malignant cells. Immunohistochemical techniques and proteomics analysis were performed to investigate the role of intercellular interaction between malignant cells and TAMs in the recurrence of malignant glioma.

**Results:**

Six subgroups of TAMs were annotated and M2‐like TAMs were found to increase in recurrent malignant glioma significantly. A pseudotime trajectory and a dynamic gene expression profiling during the recurrence of malignant glioma were reconstructed. Up‐regulation of several cancer pathways and intercellular interaction‐related genes are associated with the recurrence of malignant glioma. Moreover, the M2‐like TAMs can activate the PI3K/Akt/HIF‐1α/CA9 pathway in the malignant glioma cells via SPP1‐CD44‐mediated intercellular interaction. Interestingly, high expression of CA9 can trigger the immunosuppressive response in the malignant glioma, thus promoting the degree of malignancy and drug resistance.

**Conclusion:**

Our study uncovers the distinction of M2‐like TAMs between primary and recurrent glioma, which offers unparalleled insights into the immune microenvironment of primary and recurrent malignant glioma.

## Graphical abstract

Intercellular interaction between M2‐like TAMs and malignant cells was revealed. PI3K/Akt/HIF‐1α/CA9 activation promotes the recurrence of malignant glioma.

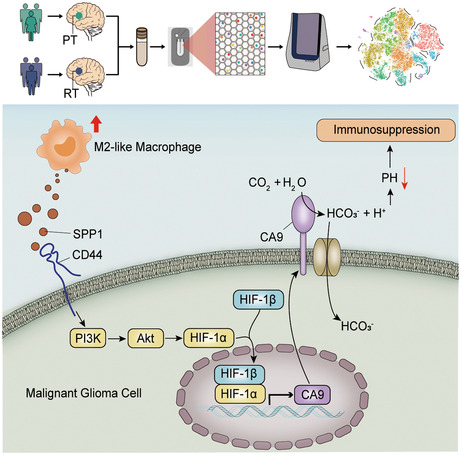

## 1 INTRODUCTION

Tumor‐infiltrating immune cells in the microenvironment of malignant glioma not only promote an immunosuppressive effect but also lead to tumor development, metastasis, and drug resistance.^1^ Tumor‐associated macrophages (TAMs) are the primary infiltrating immune cells in the immune microenvironment of malignant glioma.^2^ Typically, TAMs have two functional states: pro‐inflammatory M1 TAMs, which inhibit tumor progression, and tissue‐repair M2 TAMs, which promote tumor growth.^3^, ^4^ Further studies have found that TAMs in the immune microenvironment of malignant glioma usually polarize into M2 TAMs and exert immune‐suppressive effects in the microenvironment to promote tumorigenesis.^5^, ^6^ To further elucidate the specific mechanism, it is important to accurately analyze the immune microenvironment and compare the distinction of TAMs in the immune microenvironment between the primary and recurrent malignant glioma. However, the immune microenvironment of malignant glioma was generally studied using cell co‐culture technology, microenvironment construction technology on chips, or animal model construction which is actually different from the real physiological microenvironment in human malignant glioma.^7^, ^8^, ^9^

The emergence and development of single‐cell RNA sequencing (scRNA‐seq) provide new technical supports for exploring tumor microenvironment heterogeneity, constructing cell maps, as well as measuring cell and molecular states.^10^, ^11^ The role of different cell types in the tumor microenvironment and the specific mechanism of their influence on tumorigenesis have been revealed with the appliance of scRNA‐seq in tumor research.^12^, ^13^

Here, we performed a scRNA‐seq survey and obtained the transcriptome profiles of 23,010 cells from 3 patients with primary tumors (PT) and 3 patients with recurrent tumors (RT). We found that the proportion of immune cells in the microenvironment of the recurrent malignant glioma was much different as compared with the primary malignant glioma, especially the significantly increased proportion of M2‐like TAMs. We further combined scRNA‐seq, immunohistochemistry, and proteomic analysis to investigate the intercellular interaction in the immune microenvironment. Our experimental results suggested that M2‐like TAMs activated the PI3K/Akt/HIF‐1α/CA9 pathway in the malignant glioma cells via SPP1‐CD44‐mediated intercellular interaction to promote the recurrence of malignant glioma. Our study sheds new light on the mechanisms underlying increased drug resistance and recurrence in malignant gliomas.

## 2 MATERIALS AND METHODS

### 2.1 Patients

Six fresh tumor tissue specimens were obtained from patients who underwent glioma surgery for scRNA‐seq. Formalin‐fixed and Paraffin‐embedded (FFPE) samples, which were used to perform Immunohistochemical staining, were provided by the Department of Neurosurgery of Fujian Medical University Union Hospital. All relative ethical rules were followed in this study, which was approved by the ethics committee of Fujian Medical University Union Hospital.

### 2.2 Single‐cell RNA sequencing and data analysis

BD Rhapsody system (BD Biosciences) was used to obtain the single‐cell transcriptomic expression profile from glioma tissues in accordance with the protocol provided by the manufacturer, and the details were described in the previous study.^14^ In short, immediately after the resection by professional neurosurgeons, fresh tumor tissue specimens were rapidly transported to the lab on ice, followed by mincing, digesting, and suspending. After cell viability assessment and cell counting, the single‐cell suspension was then loaded into the BD Rhapsody cartridge, where single‐cell mRNA was captured by magnetic beads, which were then collected for cDNA synthesis by reverse transcription. cDNA was then amplified and converted into libraries for sequencing on NovaSeq6000 (Illumina) to obtain raw sequencing data processed by the BD Rhapsody analysis pipeline.

Gene‐Barcode matrices were generated and then imported into the Seurat (v3.0.2).^15^ Cells with <200 or >6344 detected genes were excluded. Using the Percentage Feature Set function of the Seurat package to calculate the mitochondria expression, low‐activity cells whose expression of mitochondria genes was >34.2% were excluded. Then, using Integrate Data in the Seurat package, we integrated data from six samples after using Find Integration Anchors to identify ‘anchors’ between different data sets. After integrating, we selected the top 30 PCA components and visualized cell clusters by the t‐distribution stochastic neighbor embedding (t‐SNE) method. To find significant deferentially expressed genes, the Wilcoxon Rank‐Sum Test was used for each cluster, compared with the remaining clusters. We used SingleR (V1.0.0) to identify the cell types by conventional marker genes. Copy number variations (CNVs) were detected using the InferCNV R package (V1.0.3) to assess accuracy of clustering. The raw count matrix was used as input data, and the immune cells and endothelial cells were used as the reference cells.^16^ To evaluate the correlation between cells in each group, we used the cor function built into R to calculate the correlation coefficient matrix. Pathway analysis was based on the KEGG database. To analyze the intercellular interactions between TAMs and malignant cells, CellPhoneDB was used to identify significant ligand‐receptor pairs within primary and recurrent samples. Based on rank value, the top 50 unique ligand‐receptor pairs were shown. Pseudotime analysis was performed using monocle 2.^17^

### 2.3 Cell culture and lentivirus construction

Human glioma U87 cells (ATCC) were cultured in Basal Medium with 10% FBS (Ausbian, 164210). Lentiviruses‐carrying short hairpin RNA (shRNA) against human hypoxia‐inducible factor 1α (HIF‐1α) were purchased from Genechem Company. The RNAi sequence targeting human HIF‐1α was GCTGACCAGTTATGATTGT. Viruses were amplified and titrated in U87 cells. Lentiviruses containing empty plasmids (vector) were used as a control. Then, total RNA was isolated from cells with TRIzol reagent (Pufei Biotech). Real‐time quantitative PCR was performed (GAPDH as an internal control). Primer pairs used are listed in Table S3.

### 2.4 Proteomic profiling

#### 2.4.1 Sample preparation

Filter‐aided sample preparation (FASP) method was employed for sample preparation as previous research described.^18^ Briefly, SDT buffer (4%SDS, 100 mM Tris–HCl, pH 7.6) (Sangon, Shanghai, China, SB0485‐500g) was added to the sample to lyse U87 cells. Then, the BCA‐assay (bicinchoninic acid) was performed for protein quantification (Beyotime, P0012S). To assess the sample quality, all samples were separated on 12.5% SDS‐PAGE gel and then stained with Coomassie Blue R‐250 (Beyotime, ST031). For digestion, we incorporated 100 mM DTT (Sangon, A620058) and 100 mM IAA (Sangon, A600539) buffer into 200 μg of protein solution for each sample in order in 30kD ultrafiltration tubes (Sartorius, VN01H22), and the filters were washed with UA buffer (8 M Urea, 150 mM Tris–HCl, pH 8.5) (BIO‐RAD, 161–0731) three times and then 0.1 M TEAB (Sangon, A510932) twice, with centrifuging at 12,500*g* for 25 min. Adding Trypsin buffer (4 μg Trypsin in 40 μL 0.1 M TEAB solution) (Promega, V5117) and leaving at 37°C for 16–18 h, the resulting peptides were collected as a filtrate after centrifuging at 12,500 *g* for 15 min.

#### 2.4.2 Tandem mass tag (TMT) labeling and LC–MS analysis

The workflow was based on previous studies.^19^, ^20^ As per manufacturer's instructions, peptide mixture was labeled by TMT 6plex Isobaric Mass Tag Labeling kit (Thermo). With the help of 1260 Infinity II HPLC (Agilent), the labeled peptides were fractionated into 10 fractions for nanoLC‐MS/MS analysis. The mobile phase and column used were described in a previous method.^21^ Detailed methods could obtain from the previous study.^22^ In brief, a Q Exactive mass spectrometer was coupled to Easy‐nLC (Thermo). We operated the mass spectrometer in positive ion mode. From the survey scan (350–1800 m/z), we select the most abundant precursor ions for HCD fragmentation. Relative parameters: resolution (70,000 at m/z 200); AGC target (3e6); Maximum IT (50 ms). Relative parameters of MS2 scans: resolution (17,500 at m/z 200); AGC target (2e5); Maximum IT (45 ms); isolation width (2 m/z).

Using the Proteome Discoverer 2.2 and MASCOT engine (Matrix Science; version 2.6), MS/MS raw files were processed. The searched database was Uniprot‐HomoSapiens, downloaded on February 2020, including 20,367 sequences.

#### 2.4.3 Immunohistochemical staining and quantification

We collected 18 FFPE samples from 9 patients, which were stained prospectively for HIF‐1α protein by Immunohistochemistry (IHC) using an anti‐human HIF‐1α monoclonal antibody (Abcam, ab51608), following the manufacturer's protocol. The antibody was diluted at a ratio of 1/100. We used the Image‐Pro Plus version 6.0 software (Media Cybernetics) to assess areas and integrated optical density (IOD) values. The HIF‐1α staining intensity was determined by the mean IOD of the digital image (magnification, ×400), indicating the relative HIF‐1α expression level. The area signal densities from 5 fields, which were randomly selected, were counted and subjected to statistical analysis.

### 2.5 Statistics

The comparison of gene expression or gene signature between two groups of cells was performed using the unpaired two‐tailed Student's *t*‐test. The cell distribution of paired RT and PT was compared using paired two‐tailed Student's *t*‐tests, and statistical significance was set at *p* < 0.05. We considered Proteins with Fold change >1.2 and *p*‐value (Student's *t*‐test) < 0.05 as differentially expressed proteins. Analyses were performed by R.

## 3 RESULTS

### 3.1 A single‐cell map of primary and recurrent malignant glioma

**FIGURE 1:**
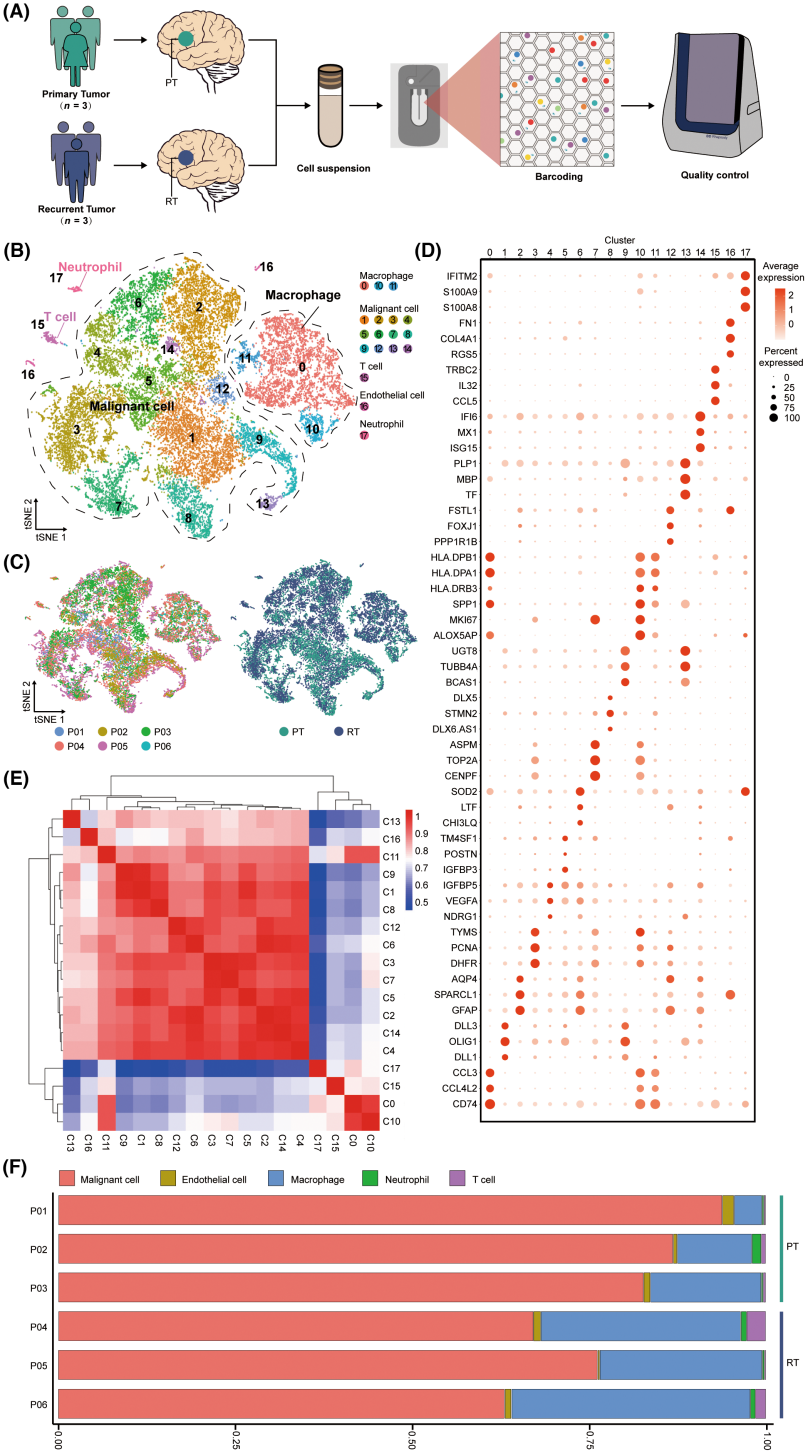
A single‐cell map of primary and recurrent malignant glioma. (A) Schematic representation of the experimental strategy. (B) t‐SNE of 23,010 high‐quality cells to visualize cell clusters based on the expression of known marker genes. (C) The t‐SNE plot, showing cell origins by color, patient origin (left panel), and PT or RT origin (right panel). (D) Dot plot showing the expression of marker genes in the indicated cell clusters. (E) The heat map indicating the correlation between different cell clusters. (F) Histogram indicating the proportion of different cell clusters in the tumor tissue of each analyzed patient.

To explore the cell characteristics in the microenvironment of primary and recurrent malignant glioma, we performed scRNA‐seq analysis on 6 malignant glioma specimens (Figure 1A; Table S1), including 3 primary and 3 recurrent specimens. Based on the counts of genes detected in each cell, a total of 23,010 cells passed initial quality control for further analysis after screening low‐quality cells with few or abnormally high numbers of genes (Figure 1A; Figure S1A–D and Table S2).

Following by removing batch effects among multiple samples, we use the Seurat software suite to perform dimensionality reduction and unsupervised cell clustering (Figure S2A,B). Distinct cell populations were identified based on the variably expressed genes (Figure S2C). After data visualization and cell annotation by t‐SNE, the results demonstrated that cells in the microenvironment of malignant glioma mainly included malignant cells, TAMs, T cells, endothelial cells, and neutrophils (Figure 1A–D; Figure S3A–G). These cell types were present in all 6 samples (Figure S4A–E).

Among immune cells, T cells and neutrophils accounted for a low proportion in each sample, while TAMs accounted for a high proportion (Figure 1F). In addition, three clusters of TAMs were sorted in our experiments, suggesting heterogeneity of TAMs in malignant glioma. We also conducted correlation analysis on all 18 clusters of cells (Figure 1E). We found a strong correlation between the cell clusters annotated as malignant cells, whereas a weak correlation between the cell clusters annotated as T cells and neutrophils. Among the cell clusters annotated as TAMs, C0 and C10 also presented a low intensity of correlation with the malignant cells, which consolidated the accuracy of cell annotation to a certain extent (Figure 1E). Interestingly, the result indicated a strong correlation of C11 and C16 with malignant cells, which may result from cell interactions in the microenvironment (Figure 1E).

### 3.2 Annotation of multiple TAMs subgroups in the immune microenvironment of malignant glioma

**FIGURE 2:**
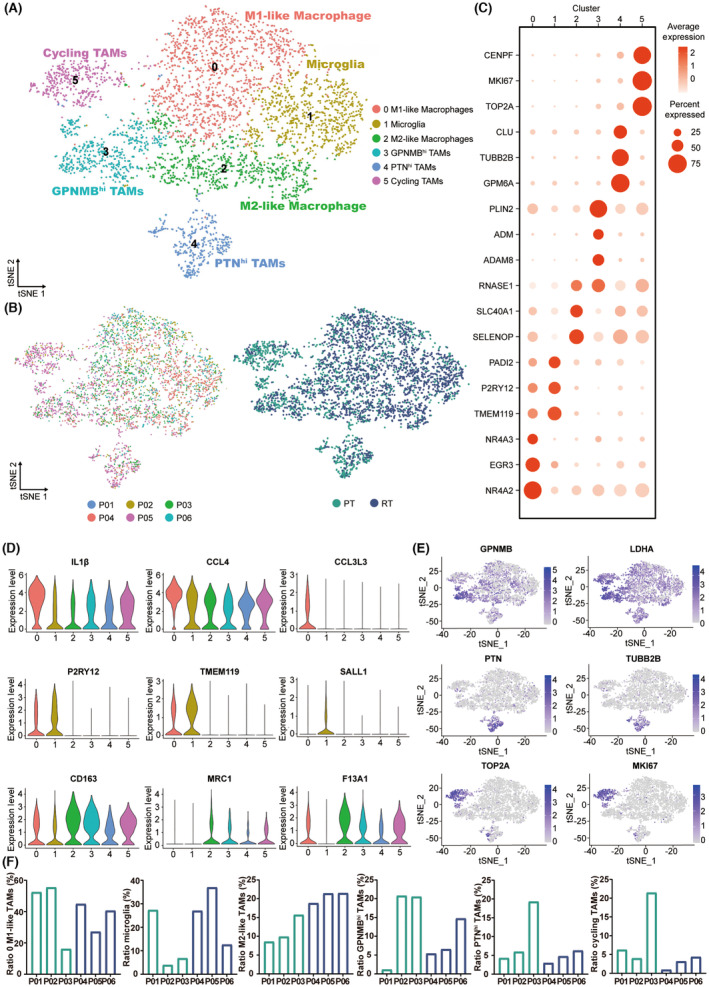
The subgroups of TAMs in the primary and recurrent malignant glioma. (A) t‐SNE projections of subgrouped TAMs, labeled in different colors. Cell type annotations are provided in the figure. Cycling TAMs refer to TAMs related to cell cycle regulation. (B) t‐SNE projections showing PT and RT TAMs, color‐coded and clustered, according to the patient (left panel) or disease state (PT/RT, right panel). (C) Dot plot showing the expression of marker genes in the indicated subgroups of TAMs. (D) Violin plot showing the expression of selected marker genes for the defined subgroups of TAMs. (E) t‐SNE visualized plot showing the expression of selected marker genes for the defined subgroups of TAMs. (F) Bar chart showing the proportion of each TAMs subgroup in PT/RT samples.

From the single‐cell map in Figure 1B and the histogram in Figure 1F, TAMs account for a large proportion of immune cells in the microenvironment of malignant glioma, and a high degree of heterogeneity was found among the TAMs clusters. To further explore the immune microenvironment of malignant glioma, a total of six subgroups (0–5 subgroups) were derived by re‐clustering analysis, as can be seen from Figure 2A–C. After analyzing the expression of differential genes in different cell subgroups, subgroup 0 was defined as M1‐like TAMs (IL‐1β, CCL4, CCL3L3) (Figure 2D; Figure S5A).^23^, ^24^ Compared with other subgroups, M1‐like TAMs exhibited significantly high expression of some inflammatory factors, suggesting that they had strong antigen‐killing and pro‐inflammatory effects (Figure S5A). Subgroup 1 was defined as activated microglia (P2RY12, TMEM119, SALL1) (Figure 2D; Figure S5A).^25^, ^26^, ^27^ Subgroup 2 was defined as M2‐like TAMs (CD163, MRC1, F13A1) (Figure 2D; Figure S5A).^23^, ^28^

Subgroups 3–5 could not be defined as the TAMs subgroups classified in the past, so we annotated them as the TAMs subgroup with high expression of a particular gene according to the differentially expressed genes and gene‐related characteristics. Although different levels of GPNMB were expressed in different subgroups of TAMs, the highest level of GPNMB expression was found in subgroup 3 (Figure 2E). Hence we defined subgroup 3 as TAMs with high expression of GPNMB (GPNMB^hi^ TAMs). The high upregulation of GPNMB expression in TAMs was associated with the poor prognosis of malignant glioma.^29^ Moreover, GPNMB^hi^ TAMs also showed differential expression of immunosuppressant‐related genes LDHA and LGALS1, as well as the poor prognostic marker LGALS3 in malignant glioma (Figure 2E; Figure S5A).^30^, ^31^, ^32^ Likewise, we annotated subgroup 4 as PTN^hi^ TAMs because of the high expression of pleiotrophin (PTN) in subgroup 4 (Figure 2E). In addition, PTN^hi^ TAMs showed a higher expression of differential genes such as TUBB2B, MARCKSL1, and C1orf61 than other TAM subgroups, which have a vital role in the formation and development of tumors (Figure S5A).^33^, ^34^, ^35^ For subgroup 5, we defined it as TAMs related to cell cycle regulation according to its high expression of TOP2A, MKI67, CENPF, CENPE, and other differential genes relevant to cell proliferation and cell cycle regulation (Figure 2E; Figure S5A).^36^, ^37^, ^38^

We then analyzed the distinctions of TAMs in the immune microenvironment between primary and recurrent malignant glioma. However, the proportion of M1‐like TAMs, activated microglia, GPNMB^hi^ TAMs, PTN^hi^ TAMs, and TAMs related to cell cycle regulation did not show consistent variation in the comparison of recurrent and primary malignant glioma (Figure 2F). Notably, the increased proportion of M2‐like TAMs in recurrent malignant gliomas compared to primary malignant gliomas was specific and consistent (Figure 2F). The result is consistent with previous studies: the proportion of M2 TAMs in recurrent malignant glioma is significantly higher than that in primary malignant glioma,^39^, ^40^ which greatly impedes the treatment of malignant glioma. However, the exact mechanism remains unclear.

### 3.3 Landscape of malignant cells in the immune microenvironment of malignant glioma

**FIGURE 3:**
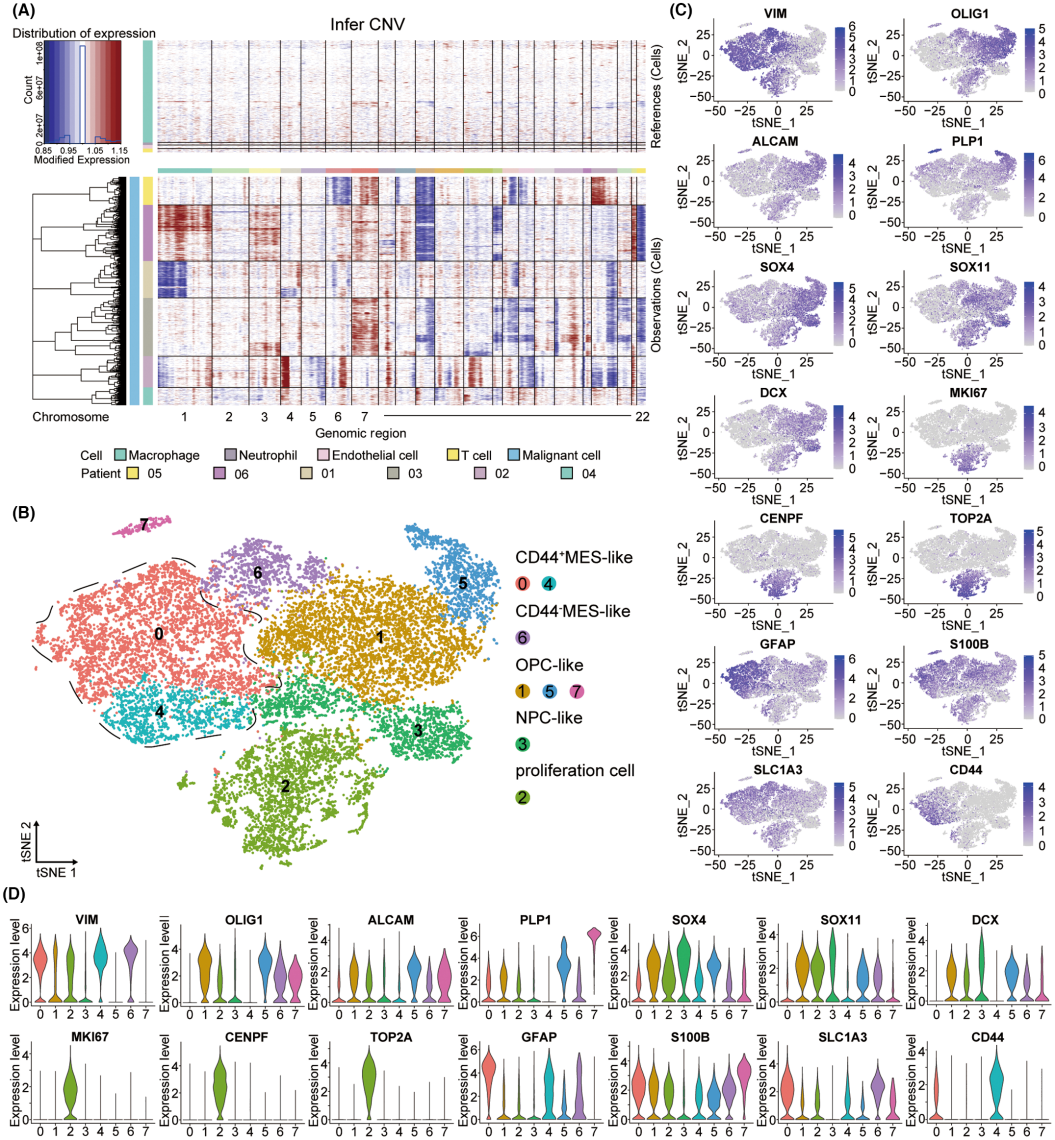
The subgroups of malignant cells in the primary and recurrent malignant glioma. (A) InferCNV plot shows significant copy number variations in the chromosomes of malignant cells. Immune cells and endothelial cells were used as references (top). The red color represents amplification and the blue represents deletion. (B) t‐SNE projections of subgrouped malignant cells, labeled in different colors. Cell type annotations are provided in the figure. Proliferation cells refer to the malignant cells related to proliferation. (C) t‐SNE visualized plot showing the expression of selected marker genes for the defined subgroups of malignant cells. (D) Violin plot showing the expression of selected marker genes for the defined subgroups of malignant cells.

As shown in Figure 1B and Figure S3B, cells with high expression of BCAN, OLIG1, GFAP, PLP1 and OLIG2 were annotated as malignant cells. To check whether all of the malignant cells were malignant cells, copy number variations (CNV) were detected to infer large‐scale copy number alterations for each cell. The heatmap revealed that CNVs accumulated in malignant cells and showed significant heterogeneity in patients (Figure 3A). Previous studies have reported that the gain of chromosome 7 and loss of chromosome 10 were the two most common genetic alterations in glioblastoma.^41^ However, not all malignant cells exhibited a significant gain of chromosome 7 and loss of chromosome 10 in our research. Such results may be caused by the fact that our samples were not composed exclusively of glioblastoma samples.

Further, we found 8 subgroups of malignant cells by re‐clustering analysis (Figure 3B). Previous studies have recapitulated the cellular states of malignant cells in malignant glioma, including neural‐progenitor‐like (NPC‐like), oligodendrocyte‐progenitor‐like (OPC‐like), astrocyte‐like (AC‐like), and mesenchymal‐like (MES‐like).^41^, ^42^, ^43^ According to expression of differential genes, the subgroups of malignant cells were annotated as follows: (1) the MES‐like malignant cells with high expression of VIM; (2) the OPC‐like malignant cells characterized with high OLIG1, ALCAM and PLP1 expression; (3) the NPC‐like malignant cells highly expressing SOX4, SOX11 and DCX; (4) the malignant cells related to proliferation expressing the proliferation markers MKI67, CENPF, TOP2A (Figure 3B–D).^42^, ^44^, ^45^ We found that markers of AC‐like cells (GFAP, S100B, SLC1A3) were widely expressed in each subgroup, so none of the subgroups was annotated as AC‐like malignant cells (Figure 3C,D).^42^ Interestingly, we noticed that another mesenchymal marker CD44 was highly expressed only in clusters 0 and 4 but not in cluster 6 (Figure 3C,D).^46^ Based on this, we defined cluster 0 and cluster 4 as CD44^+^ MES‐like malignant cells and cluster 6 as CD44^−^ MES‐like.

### 3.4 Dynamic gene expression profiles during recurrence of malignant glioma

**FIGURE 4:**
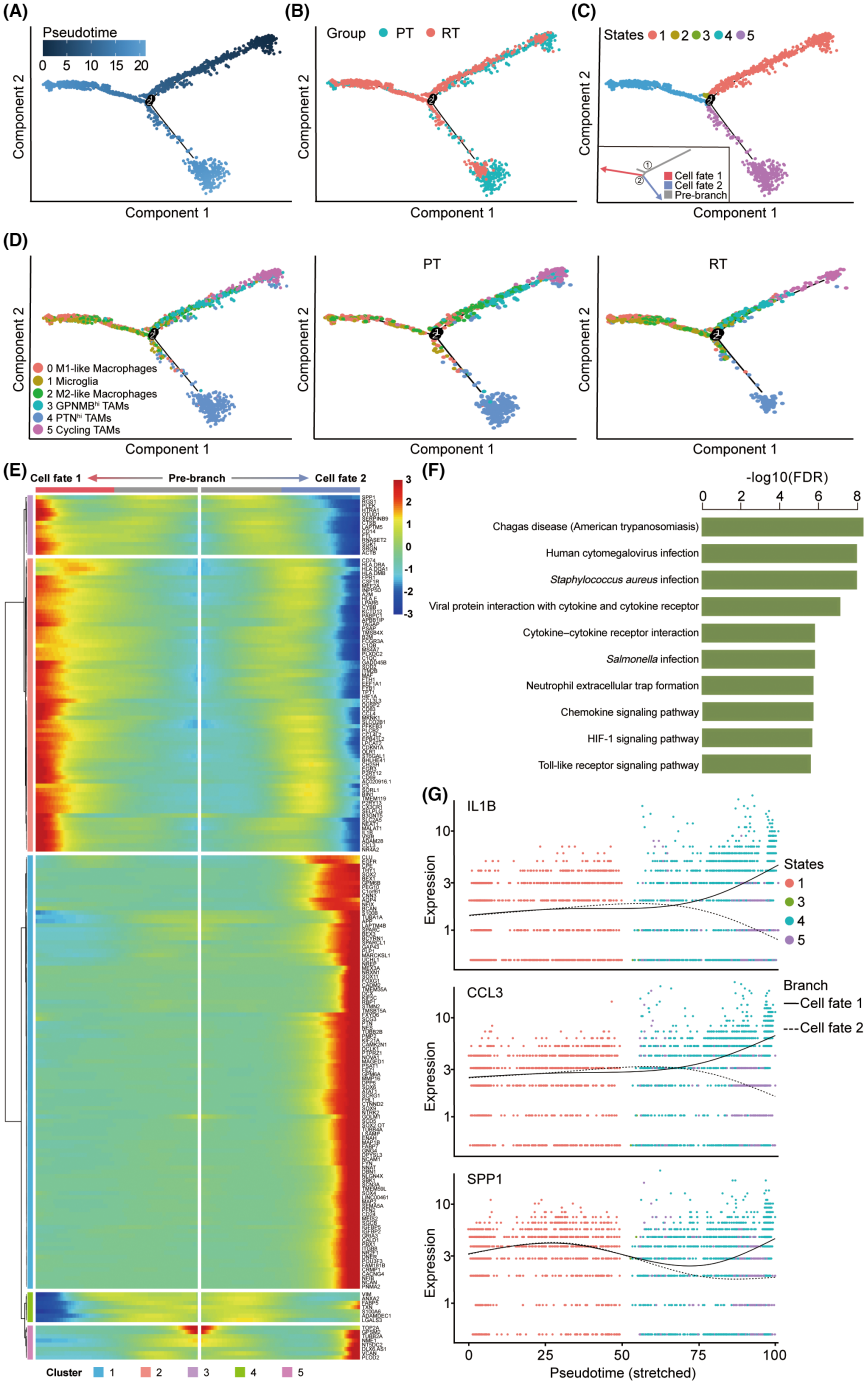
Gene expression profiles during recurrence of malignant glioma. (A) The pseudotime trajectory of TAMs inferred by analysis with Monocle2. Color key from dark to bright indicates cancer progression from the early to the late stage. (B) The pseudotime trajectory showing PT and RT TAMs. (C) The pseudotime trajectory of TAMs. TAMs on the pseudotime trajectory are colored by states. Cell fates (including pre‐branch) are provided in the figure. (D) The pseudotime trajectory of subgrouped TAMs, labeled in different colors. Cell type annotations are provided in the figure. (E) Top 200 differentially expressed genes with expression levels that changed the most over the pseudotime trajectory were divided into 5 gene clusters based on their expression trend. Cell fate 1 and cell fate 2 trajectories (including pre‐branch) are shown on the right and left, respectively. Color key from blue to red indicates relative expression levels from low to high. (F) Histogram showing the selected 10 most significantly enriched specific pathways in the gene cluster 2. (G) Expression patterns of representative differentially expressed genes during recurrence of malignant glioma.

Pseudotime analysis was performed to generate the pseudotime trajectory of TAMs during the recurrence of malignant glioma. Distribution of TAMs from 6 patients was yielded in the pseudotime trajectory that can indicate the change of the immune microenvironment during the recurrence of malignant glioma (Figure 4A–C; Figure S5B). Along the pseudotime trajectory, the percentage of TAMs in the recurrent malignant glioma increased in the late‐stage cell population, which verified the reliability of the pseudotime analysis (Figure 4B). The result indicated that most M1‐like TAMs, activated microglia, and M2‐like TAMs were mainly located at cell fate 1, while PTN^hi^ TAMs were mainly located at the end of cell fate 2 (Figure 4D). Noticeably, the M2‐like TAMs in the primary malignant glioma were mainly located at the early‐middle‐stage of the trajectory, while those in the recurrent malignant glioma were mainly located at the middle‐last‐stage (Figure 4D). This difference did not appear in the other 5 subgroups, which confirmed that M2‐like TAMs are the key factor in the recurrence process of malignant glioma.

We analyzed the gene expression along the pseudotime trajectory and constructed dynamic gene expression profiles during the recurrence of malignant glioma (Figure 4E). Kyoto Encyclopedia of Genes and Genomes (KEGG) analyses were performed to predict the biological roles and potential signaling pathways of these differentially expressed genes. Along the trajectory of cell fate 1, the expression of genes related to cytokine‐cytokine receptor interaction, chemokine signaling pathway, and HIF‐1 signaling pathway was upregulated, such as IL‐1B and CCL3 (Figure 4F,G). Previous studies have shown that these pathways have a critical role in cancer development,^47^, ^48^ suggesting that TAMs in cell fate 1 are the chief culprit of the recurrence. Interestingly, we noticed that the expression of cytosol and protein binding‐related genes, such as secreted phosphoprotein 1 (SPP1), also increased or fluctuated in TAMs of cell fate 1, which indicated a strong intercellular interaction between TAMs and malignant cells (Figure 4E,G). Previous studies have suggested that SPP1 can regulate metastasis and invasion of the tumor as a part of the extracellular matrix‐receptor interactions.^49^ A recent single‐cell study of glioma also revealed that SPP1/CD44‐mediated intercellular interaction between macrophages and cancer cells plays a critical role in glioma progression.^50^ Together, these results demonstrate the distribution of M2‐like TAMs on the pseudotime trajectory and the dynamic gene expression profiling during the recurrence of malignant glioma and indicate that the up‐regulation of several cancer pathways as well as the intercellular interaction‐related genes is associated with recurrence of malignant glioma.

### 3.5 PI3K/Akt pathway activated by the intercellular interaction promotes recurrence

**FIGURE 5:**
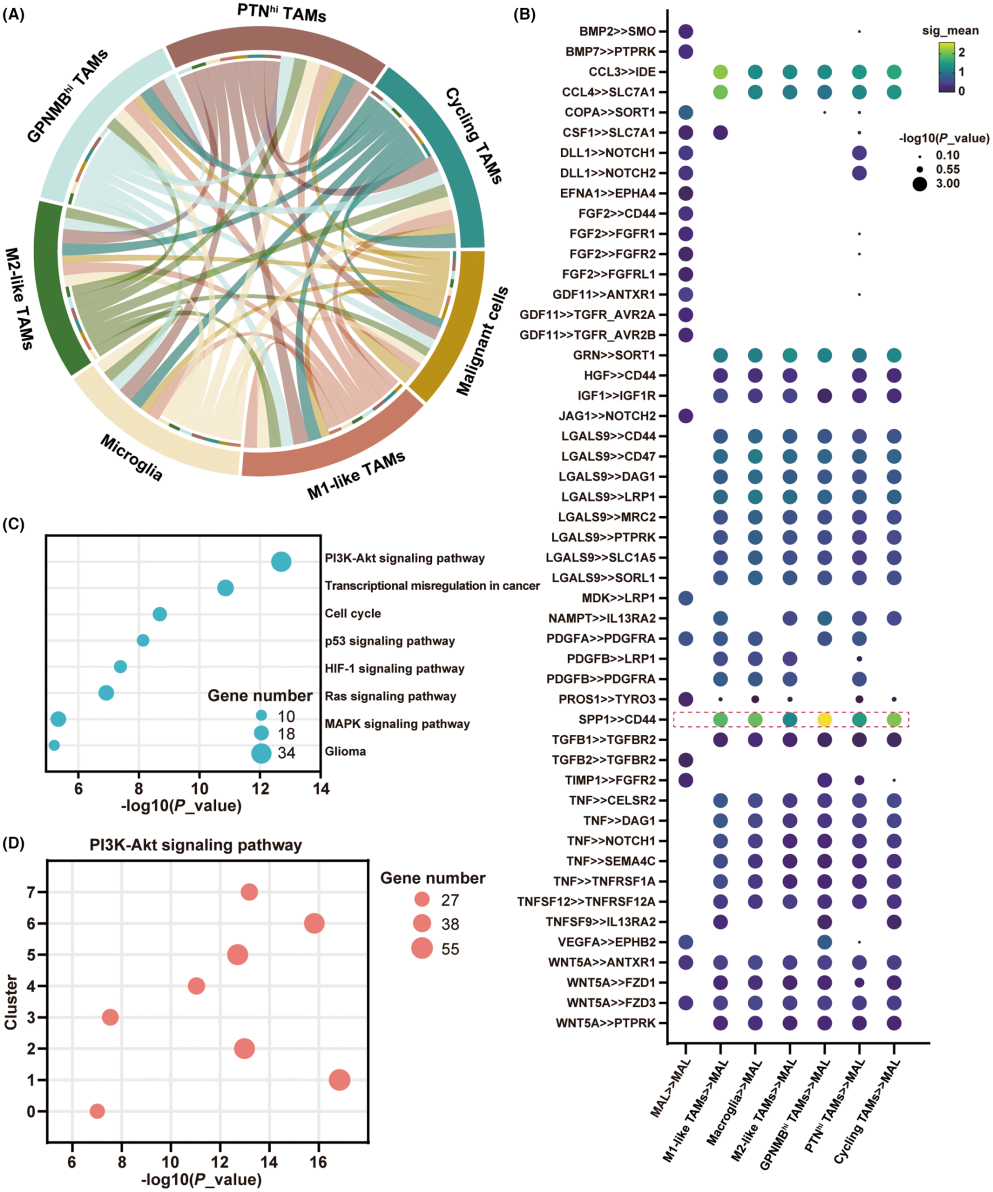
M2‐like TAMs play a crucial role in the recurrence of malignant glioma. (A) Circos Plot showing the intercellular interaction between malignant cells and 6 defined subgroups of TAMs in the microenvironment of malignant glioma. (B) Bubble plot showing ligand‐receptor pairs between TAMs and malignant cells. Bubble colors refer to communication probabilities, whereas Bubble sizes represent the calculated *p*‐values. (C) Bubble plot showing the selected 9 most significantly enriched specific pathways in the malignant cells. (D) Bubble plot showing the enrichment of PI3K/Akt signaling pathway in 8 subgroups of malignant cells.

Cellular association analysis was performed to study the intercellular interaction between TAMs and malignant cells. Consistent with the result of pseudotime analysis, active interactions exist between malignant cells and different subgroups of TAMs (Figure 5A).

We then investigated the intercellular interactions mediated by ligand‐receptor interactions. We observed that SPP1 expressed by TAMs could bind to the CD44 receptor on malignant cells (Figure 5B). CD44 is a cell adhesion molecule that plays an important role in tumor progression and metastasis due to its binding to extracellular matrix components, including SPP1.^51^ Although all subgroups can affect malignant cells through SPP1‐CD44‐mediated intercellular interactions, the role of M2‐like TAMs in SPP1‐CD44‐mediated intercellular interactions is most likely a key factor in promoting recurrence progression, given that only the proportion of M2‐like TAMs is significantly increased in the immune microenvironment of recurrent malignant gliomas.

Furthermore, the KEGG analysis was used for exploring the expression of differential genes in malignant cells affected by M2‐like TAMs. The result indicated that several cancer pathways, including PI3K/Akt and P53, were significantly up‐regulated (Figure 5C). We found that the PI3K/Akt pathway‐associated genes were significantly expressed in each subgroup (Figure 5D). Notably, recent studies have also suggested that CD44 can activate the PI3K/Akt pathway.^52^

Taken together, our experimental results offered new insights into understanding the intercellular interaction between M2‐like TAMs and malignant cells.

### 3.6 Activation of the PIK3/Akt/HIF‐1α/CA9 pathway in malignant cells can induce the immunosuppressive effect in the microenvironment

**FIGURE 6:**
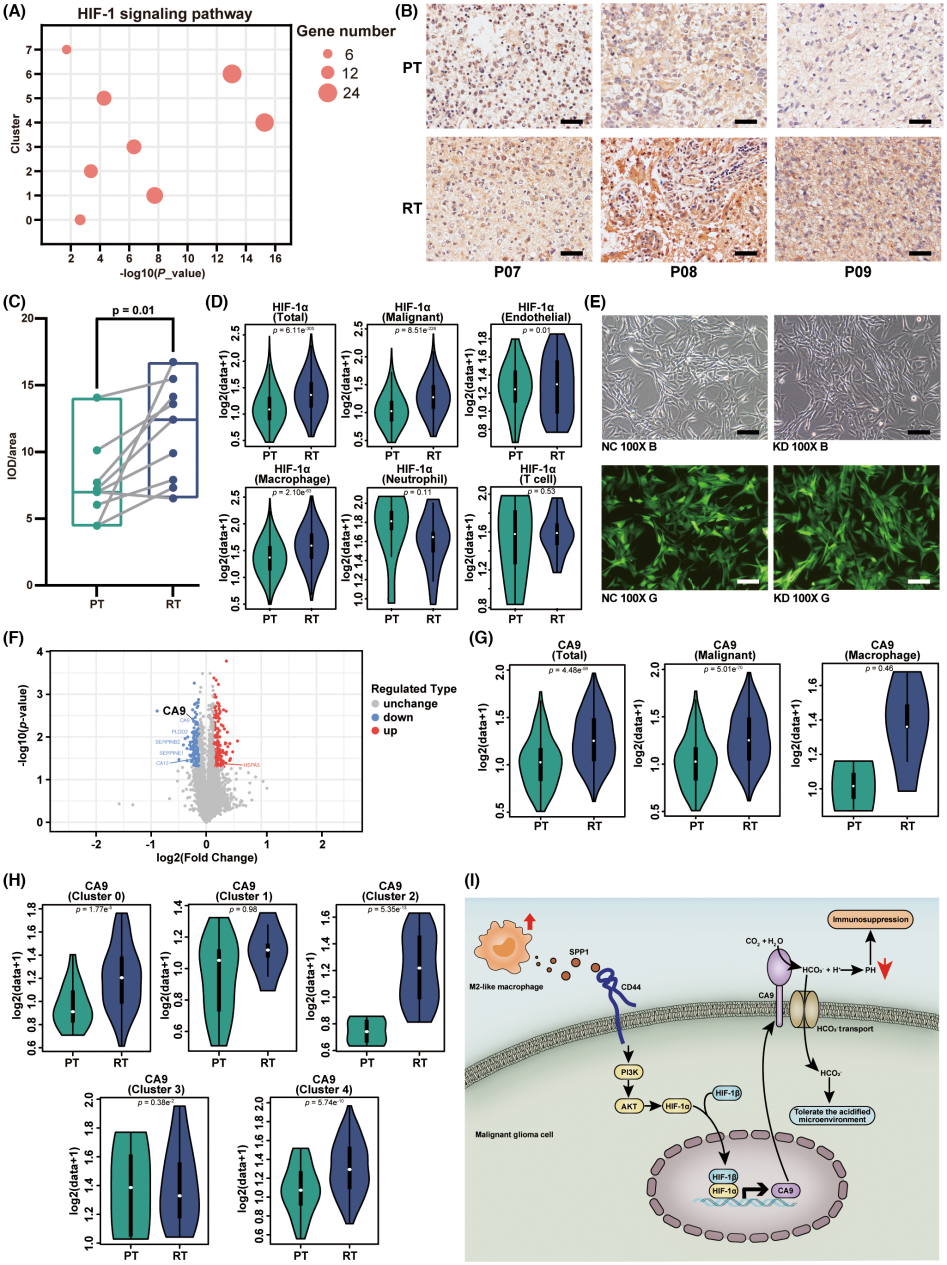
HIF‐1α/CA9 pathway upregulated in the recurrent malignant glioma. (A) Bubble plot showing enrichment of the HIF‐1 signaling pathway in 8 subgroups of malignant cells. (B) IHC staining of HIF‐1α antibody, showing the expression of HIF‐1α in paired PT and RT malignant glioma tissue. Scale bar, 10 μm. (C) Boxplot illustrating the expression of HIF‐1α in 9 matched PT and RT pairs. The *p*‐value was calculated by Student's *t*‐test. (D) Violin plot showing the HIF‐1α level of different cell types in PT and RT samples. The *p*‐value was calculated by Student's *t*‐test. (E) The microscopy image of U‐87 cell line after transfection using lentivirus. Magnification: 100×. NC: negative control. KD: knock down. B and G represent the bright field and green fluorescent field, respectively. Scale bar, 10 μm. (F) Volcano plot shows the differentially expressed genes in the KD group, compared with the NC group. Names of the most significant genes are indicated in the plot. (G) Violin plot showing the CA9 level of different cell types in the PT and RT samples. The *p*‐value was calculated by Student's *t*‐test. (H) Violin plot showing the CA9 level of each malignant cell subgroup in the PT and RT samples. The *p*‐value was calculated by Student's *t*‐test. (I) Schematic diagram of the intercellular interaction between M2‐like TAMs and malignant glioma cells in recurrent malignant glioma.

Previous studies have revealed that PI3K/Akt pathway can regulate the downstream factor HIF‐1α.^53^ As shown in Figure 5C, the HIF‐1α pathway‐associated genes were highly expressed in the malignant cells. We performed the KEGG enrichment analysis to determine whether the HIF‐1α pathway can be activated in each subgroup of malignant cells. We found that the HIF‐1α pathway‐associated genes were highly expressed in each subgroup (Figure 6A).

Furthermore, we used immunohistochemical techniques and proteomics analysis to study the effect of the PI3K/Akt pathway and its downstream factor HIF‐1α on recurrent malignant glioma. The results showed that the level of HIF‐1α was significantly increased in the tumor tissues of recurrent malignant glioma (Figure 6B,C). Consistent with the immunohistochemical results, the data of the scRNA‐seq analysis also suggested that the level of HIF‐1α in each cell cluster in the microenvironment of recurrent malignant glioma was generally higher than that in primary malignant glioma (Figure 6D). We then compared the HIF‐1α level of each cell type in the primary and recurrent malignant glioma. The results suggested that the increase of the HIF‐1α level in the microenvironment of recurrent malignant glioma was attributed to the malignant cells, TAMs, and endothelial cells (Figure 6D). Notably, the expression level of HIF‐1α in neutrophils and T cells is not changed significantly (Figure 6D).

In further experiments, we knocked out HIF‐1α in the malignant cells to study how the change in the level of HIF‐1α influences the microenvironment of malignant glioma (Figure 6E). Additionally, we performed proteomic and GO analysis to screen downstream differential proteins. The results suggested that carboxyanhydrase 9 (CA9) was the downstream protein of HIF‐1α and was related to the immune microenvironment of malignant glioma (Figure 6F; Figure S6A–E).

Notably, CA9 is a typical protein that can catalyze carbon dioxide to synthesize bicarbonate and proton in cells, which allows tumor cells to maintain a neutral pH even in the acidified microenvironment.^54^ Thus the tumor cells obtained the ability to tolerate the acidic microenvironment and facilitate the progression of tumor invasion and metastasis.^55^ However, other cells cannot tolerate the acidified microenvironment. When the microenvironment is at low pH, antitumor effectors (such as T cells and NK cells) tend to be inactive, while immunosuppressive components (such as bone marrow cells and regulatory T cells) could be activated by acidity to promote tumor growth and block the antitumor immune response.^56^

It is noted that malignant glioma cells can use CA9 to tolerate acidified microenvironment, while immune cells with tumor‐suppressive effects are useless for the tumor inhibition effects due to the acidic environment. The acidified microenvironment also leads to increased drug resistance of malignant glioma and a poor prognosis for the patients. The data based on the scRNA‐seq analysis also confirm the above statements. ScRNA‐seq analysis showed that the CA9 level significantly increased in the recurrent malignant glioma tumor cells compared with that in the primary malignant glioma (Figure 6G). Unlike the tumor cells, there was no significant increase in the CA9 level in other cell types (Figure 6G). In particular, we observed that the variation of CA9 expression differed in different malignant cell subgroups. In the recurrent samples, the expression of CA9 were remarkably elevated in CD44^+^ MES‐like subgroups and proliferation subgroup, which indicated an enhanced tolerance of acidified microenvironment (Figure 6H). However, cluster 5–7 malignant cells were too few to perform a comparison of the CA9 expression in them. The high expression level of CA9 is responsible to trigger the immunosuppressive response in the malignant glioma, thus promoting the degree of malignancy and drug resistance of recurrent malignant glioma (Figure 6I).

## 4 DISCUSSION

Although TAMs in the immune microenvironment play an important role in drug resistance and the recurrence of malignant glioma, the specific mechanism is still not fully understood. In this work, we performed experiments to systematically study the distinction of TAMs between primary and recurrent malignant glioma. Using the scRNA‐seq techniques, we have mapped the immune microenvironment of malignant glioma and classified all cells into five types. TAMs were analyzed in‐depth for identifying different subgroups and their potential influence on tumorigenesis and tumor growth. Moreover, according to the previous studies, malignant cells were derived and annotated as MES‐like malignant cells, OPC‐like malignant cells, NPC‐like malignant cells and malignant cells related to proliferation.^41^, ^42^ In this study, we have utilized the actual immune microenvironment of malignant glioma to circumvent the shortcomings of previous studies. The results provide a theoretical and experimental basis for analyzing the poor prognosis of recurrent malignant glioma.

The focus of this study was to investigate the distinctions of M2‐like TAMs in the immune microenvironment between primary and recurrent malignant glioma and their influence on the evolution of malignant glioma. Interestingly, the proportion of M2‐like TAMs increased in the recurrent malignant glioma. Moreover, the dynamic gene expression profiles showed the increase of SPP1 in TAMs of cell fate 1 containing M1‐like TAMs, activated microglia, and M2‐like TAMs. Previous research has revealed a critical role of macrophage‐mediated SPP1/CD44 signaling in glioma progression.^50^ However, it primarily focused on the evolution of tumor cells and did not study the change of TAMs affected by the activation of SPP1/CD44 signaling. Considering its inadequacy, we explored the effect of SPP1‐CD44‐mediated intercellular interactions on downstream pathways and probed into how the TAMs change as well as their influence on the microenvironment of malignant glioma. Noteworthily, we found that the M2‐like TAMs indeed activate PIK3/Akt pathway in the malignant glioma cells via SPP1‐CD44‐mediated intercellular interactions, which promotes the growth and metastasis of tumor cells. This is responsible for the increased drug resistance, frequent relapse, and poor prognosis of recurrent malignant glioma. The intensive intercellular interactions between malignant cells and TAMs in our research also verified the forward feedback of the interplay between tumor cells and macrophages in promoting tumor progression.^57^ The mechanism of intercellular interactions we stated has successfully elucidated how the increased proportion of M2‐like TAMs in the immune microenvironment results in the difficulty of clinical treatment for patients with malignant glioma. It also provides new avenues for solving this challenging problem.

Finally, we found that the activation of the PIK3/Akt pathway could further upregulate the downstream HIF‐1α/CA9 pathway, which was the key to the immunosuppressive effect in the microenvironment. Notably, the CA9 expression level remarkably elevated in CD44^+^ MES‐like subgroups and proliferation subgroup. The HIF‐1α/CA9 pathway provides a new therapeutic target for the treatment of malignant glioma. The inhibition of CA9 expression reduces the tolerance of the tumor cells towards an acidic environment and prevents the invasion and metastasis of malignant glioma. Moreover, it can reduce the acidification degree of the microenvironment and improve the tumor‐suppressive effect of immune cells. Previous studies have shown that tumor‐infiltrating immune cell‐associated long non‐coding RNA (lncRNAs) are also implicated in cancer immunity regulation and the lncRNA IGFL2‐AS1 can mediate the inhibition of HIF‐1α degradation thus increase CA9 expression.^58^, ^59^, ^60^ Therefore, intervention of CA9 expression through lncRNAs may be a novel research direction to inhibit the progression of malignant glioma.

However, molecular features of tumor samples used in our study, such as IDH and 1p/19q status, may affect the states and proportion of malignant cell subgroups.^61^ Previous studies have associated the molecular features of glioma with clinical classification. Patients with glioma without IDH mutant or 1p/19q co‐deleted had worse outcomes.^62^, ^63^, ^64^ In addition, tumors with different IDH and 1p/19q status undergo distinct cell‐state changes at recurrence, which may interfere with the comparison of the microenvironment between primary gliomas and recurrent gliomas. Prior research has shown that IDH wild‐type gliomas had significantly higher levels of MES‐like malignant cells and M2 macrophage than IDH mutant gliomas.^42^, ^61^, ^65^ While in IDH mutant gliomas, tumors with 1p/19q co‐deletion exhibited significantly higher levels of NPC‐like malignant cells and M2‐related TAMs and lower levels of OPC‐like malignant cells than tumors without 1p/19q co‐deletion.^61^ Consequently, intercellular interactions between MES‐like malignant cells and M2‐like TAMs may contribute more to the recurrence of IDH wild‐type gliomas, which may further result in a more serious tumor‐suppressive effect of immune cells due to the increase of CA9 in the IDH wild‐type gliomas. Nevertheless, our study reveals broad differences of TAMs and their impact on malignant cells between primary gliomas and recurrent gliomas.

In summary, we have constructed single‐cell maps to understand the distinctions of TAMs in the immune microenvironment between the primary and recurrent malignant glioma using scRNA‐seq techniques. By combining the results of differential gene enrichment analysis, we proposed the specific mechanisms of how M2‐like TAMs affect tumor cells and provided new insights into the mechanism of increased drug resistance and poor prognosis of recurrent malignant glioma. We demonstrated that M2‐like TAMs activate the PI3K/Akt/HIF‐1α/CA9 pathway in the malignant glioma cells via SPP1‐CD44‐mediated intercellular interaction to promote the recurrence of malignant glioma, which revealed the mechanism of increased drug resistance in recurrent malignant glioma to provide new targets for the drug development.

## AUTHOR CONTRIBUTIONS

**Guiting You:** Conceptualization, Formal analysis, Validation, Writing‐original draft. **Zhenyu Zheng:** Conceptualization, Formal analysis, Project administration, Writing‐original draft. **Yulong Huang:** Data curation, Methodology, Software, Visualization, Writing‐original draft. **Guifen Liu:** Data Curation, Supervision, Writing‐review and editing. **Wei Luo:** Methodology, Visualization. **Jianhuang Huang:** Resources, Methodology. **Longjin Zhuo:** Resources, Investigation. **Binghua Tang:** Formal analysis, Investigation. **Shunyi Liu:** Formal analysis, Investigation. **Caihou Lin:** Conceptualization, Methodology, Funding acquisition, Writing‐review and editing.

## CONFLICT OF INTEREST STATEMENT

The authors declare that they have no known competing financial interests or personal relationships that could have appeared to influence the work reported in this paper.

## Supporting information


Appendix S1

## Data Availability

The data that support the findings of this study are openly available in CNSA at https://db.cngb.org/cnsa.
