# Supplementary material for: scRNA‐seq and proteomics reveal the distinction of M2‐like macrophages between primary and recurrent malignant glioma and its critical role in the recurrence
Source: CNS Neurosci Ther. 2023 May 17;29(11):3391–405. doi: 10.1111/cns.14269 (PMC10580349; doi:10.1111/cns.14269)
Supplement: Supplementary file 1 — Appendix S1 [file CNS-29-3391-s001.docx]

**scRNA-Seq and Proteomics Reveal the Distinction of M2-like Macrophages Between Primary and Recurrent Malignant Glioma and its Critical Role in the Recurrence**

**
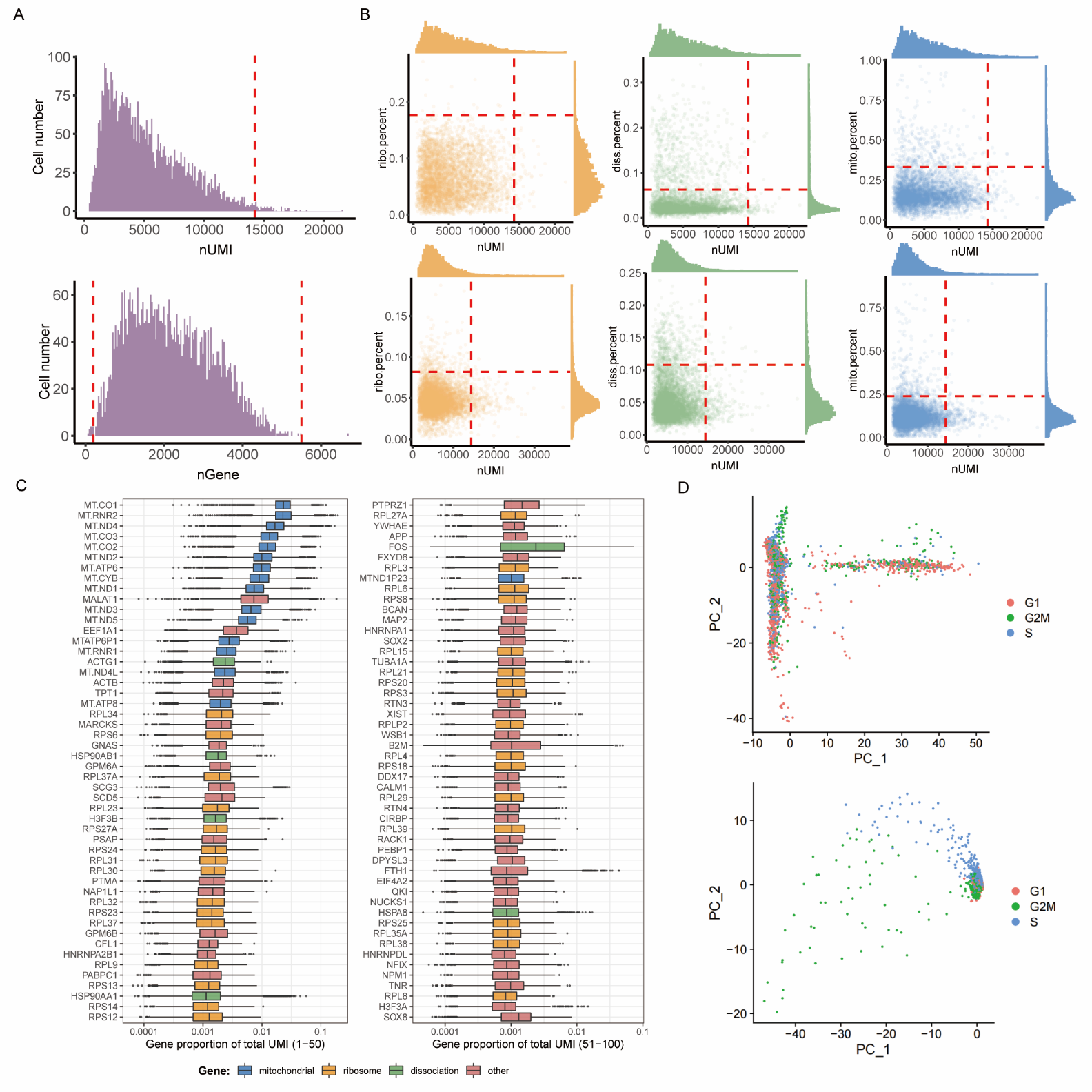
**

**Figure S1. Data Preprocessing and Normalization. Related to Figure 1.**

(A) UMI (top) and gene (bottom) distribution statistics and filtering threshold calculation of sample P01.

(B) Mitochondrial (blue), ribosomal (orange), and dissociated (green) gene proportion of samples P01 (upper) and P02 (lower) and filtration threshold.

(C) The 100 genes with the highest detection rate among the detected genes.

(D) Scatter plot of cell cycle distribution. According to the expression of all genes (upper) and cell cycle genes (lower), the cell cycle score of each cell is calculated to judge the cell cycle of each cell, and the cell cycle effect is evaluated by PCA visual data.

**
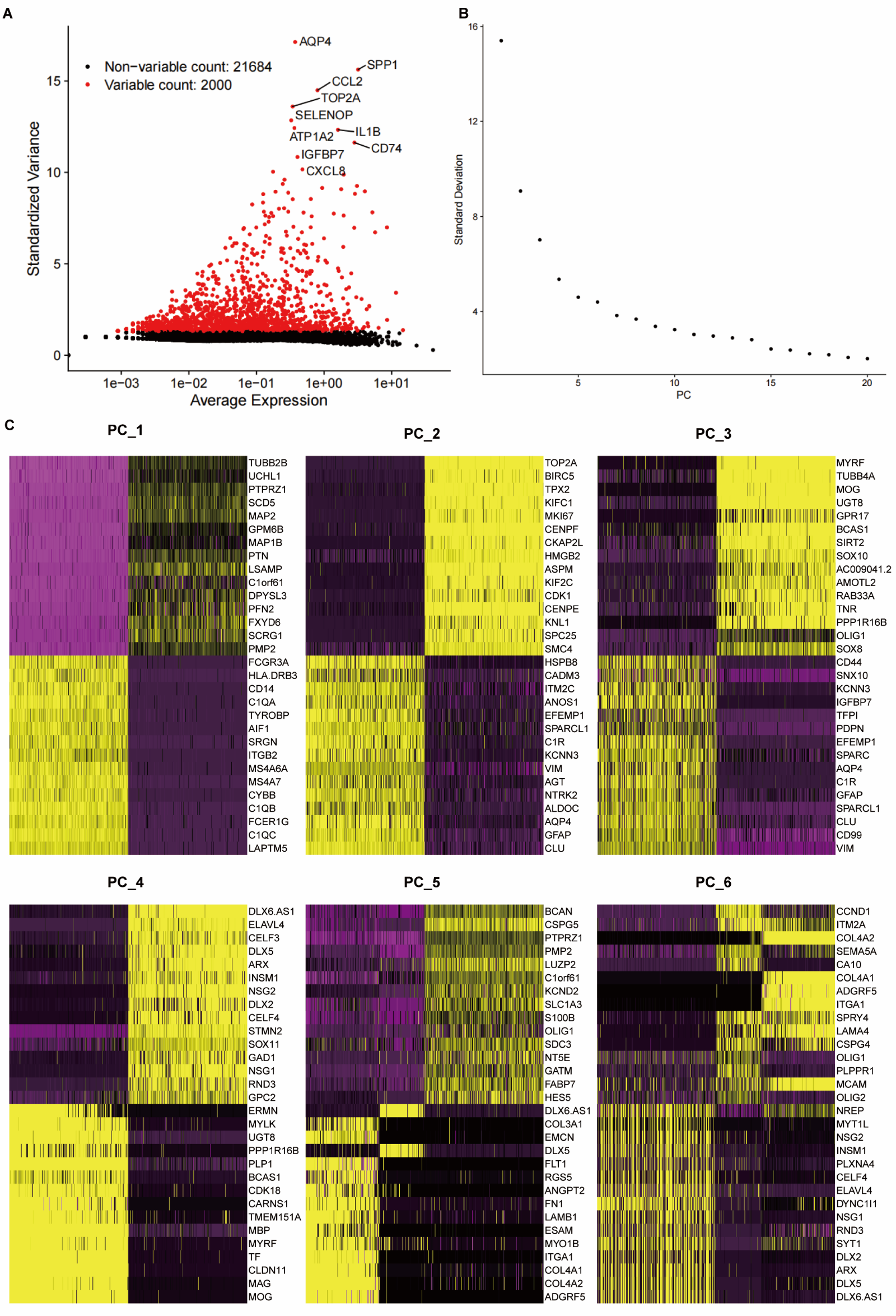
**

**Figure S2. Multi-sample Integration Analysis and PCA Dimensionality Reduction. Related to Figure 1.**

(A) The top 2,000 variant genes of sample P01. Each sample identified the top 2,000 genes with the most significant variation based on the average value and dispersion (variance/mean) of all genes for later integration analysis.

(B) Distribution of contribution under the first 20 dimensions of PCA.

(C) Heat map of marker genes in the first six dimensions of PCA. Horizontal and vertical lines represent genes and cells, respectively.

**
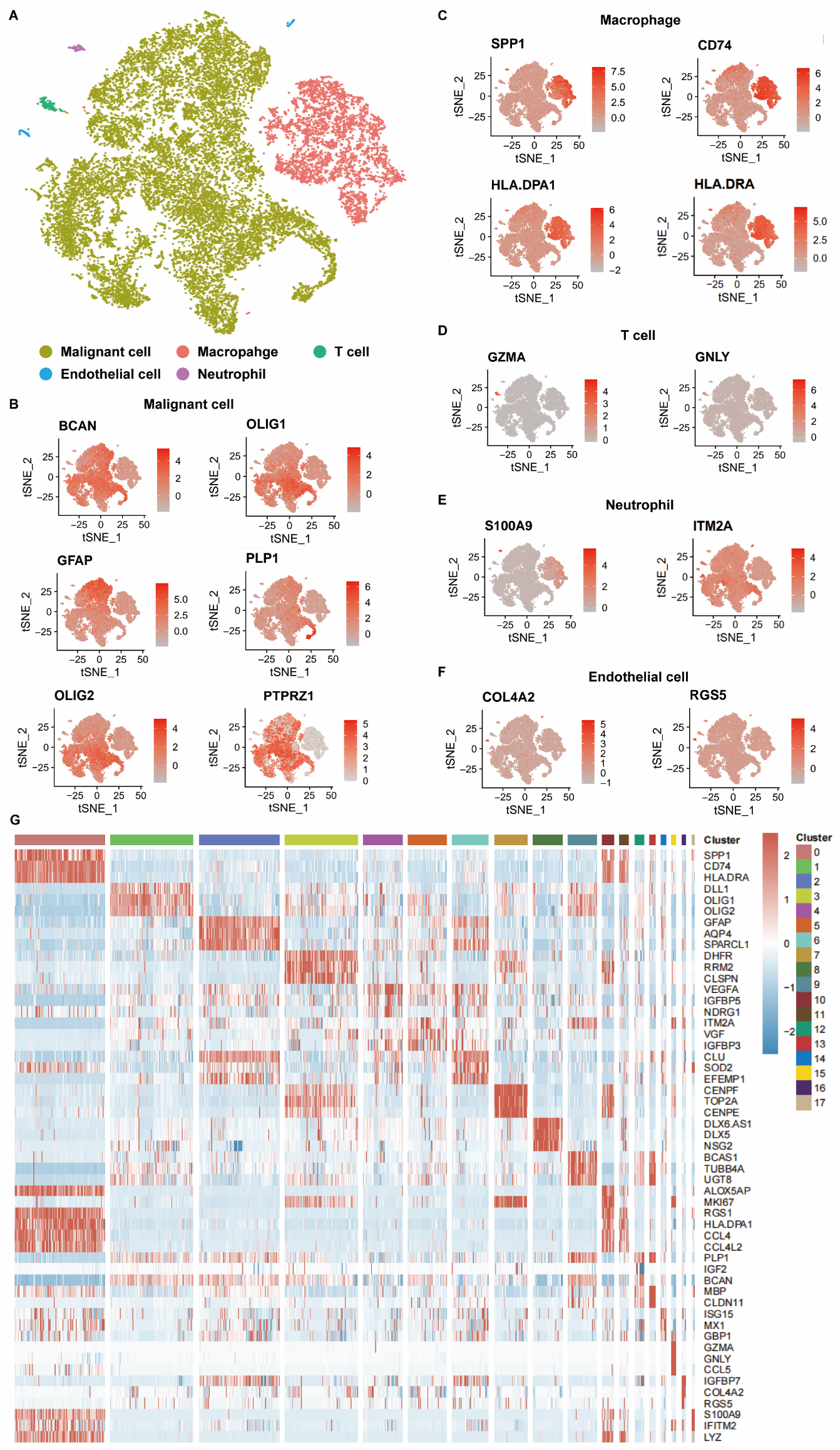
**

**Figure S3. Marker gene analysis and cell-type annotation. Related to Figure 1.**

(A) All cells were visualized by t-SNE according to cell type.

(B) Marker genes of malignant cells (BCAN, OLIG1, GFAP, PLP1, OLIG2, PTPRZ1).

(C) Marker genes of macrophages (SPP1, CD74, HLA. DPA1, HLA.DRA).

(D) Marker genes of T cells (GZMA, GNLY).

(E) Marker genes of neutrophils (S100A9, ITM2A).

(F) Marker genes of endothelial cells (COL4A2, RGS5).

(G) The heat map shows all clusters selected top 3 genes. The marker gene is in the horizontal row and the cells in the vertical row. The color bar above represents different clusters.

**
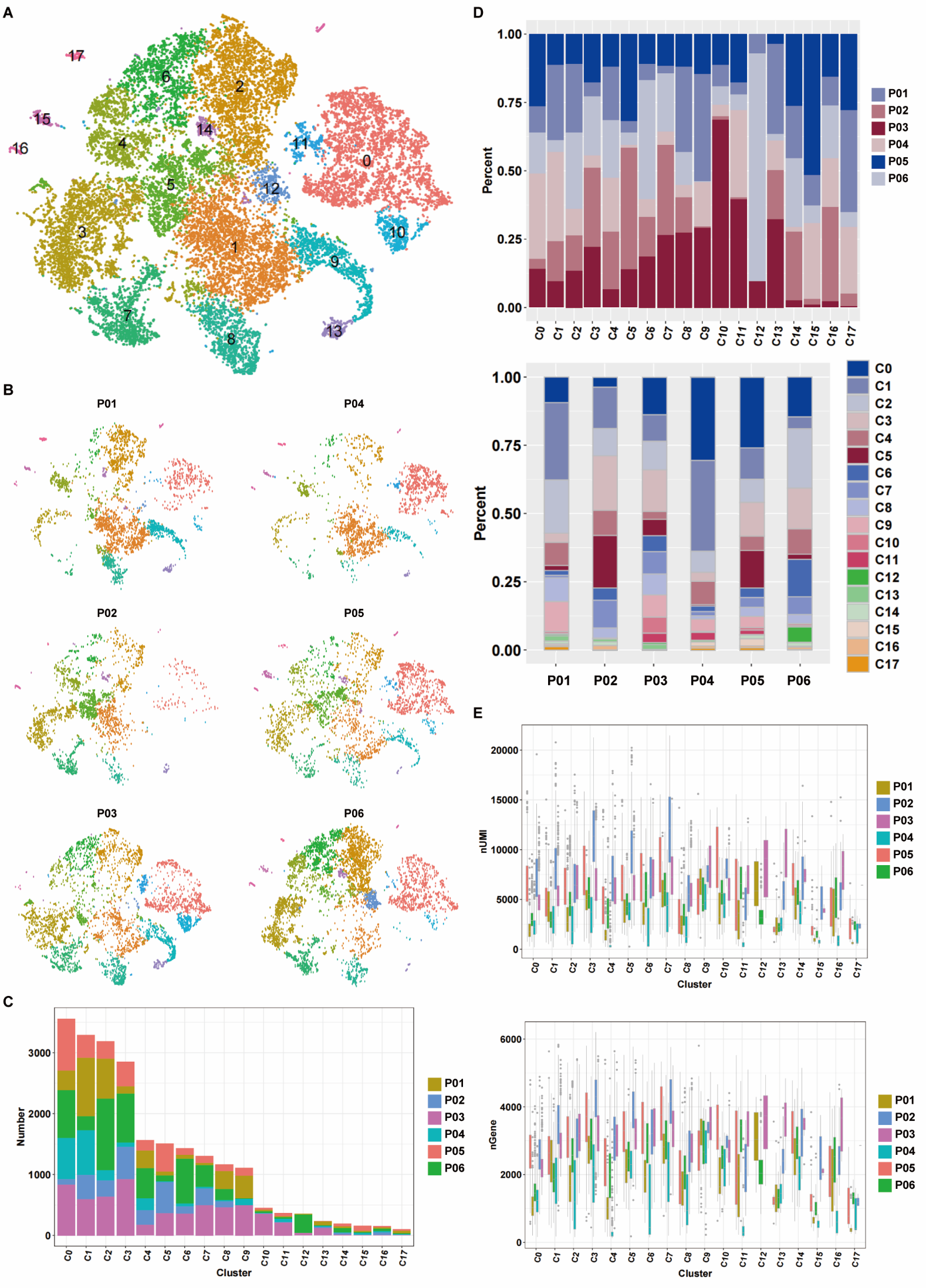
**

**Figure S4. Multi-sample comparative analysis. Related to Figure 1.**

(A) t-SNE visualization of all samples based on cluster.

(B) t-SNE visualization of each sample based on cluster.

(C) The number of cells within each cluster in different samples.

(D) Columnar stacking chart of cell-type proportion of each sample.

(E) Distribution of different cluster transcripts expression abundance (top) and gene number (bottom).

**
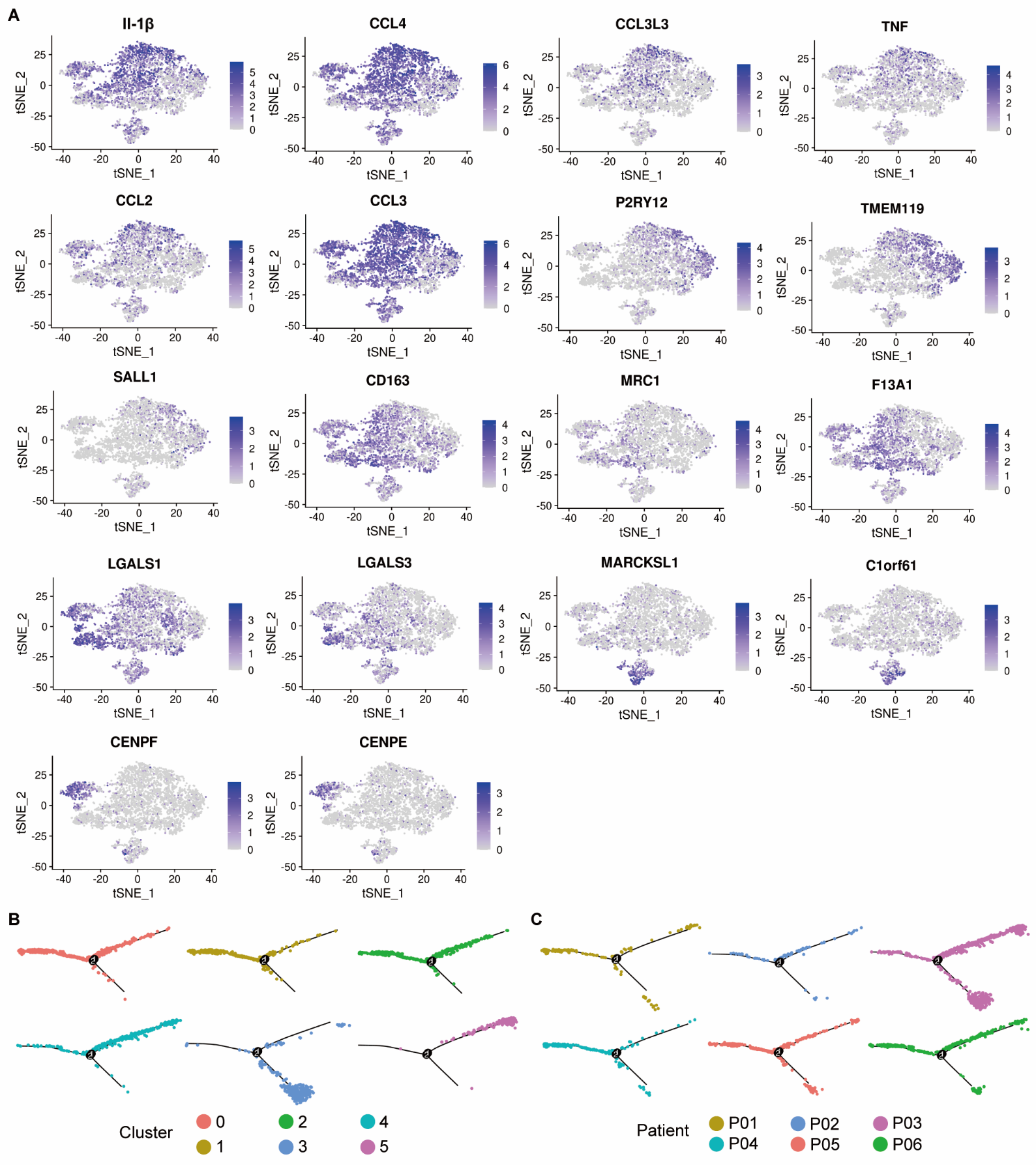
**

**Figure S5. The Subgroups of TAMs in the Primary and Recurrent Malignant Glioma. Related to Figure 2 and Figure 4.**

(A) t-SNE plot showing the expression levels of marker genes, defined for macrophage subtypes.

(B) Trajectory analysis colored by TAMs subgroups from total samples.

(C) Trajectory analysis colored by samples.

**
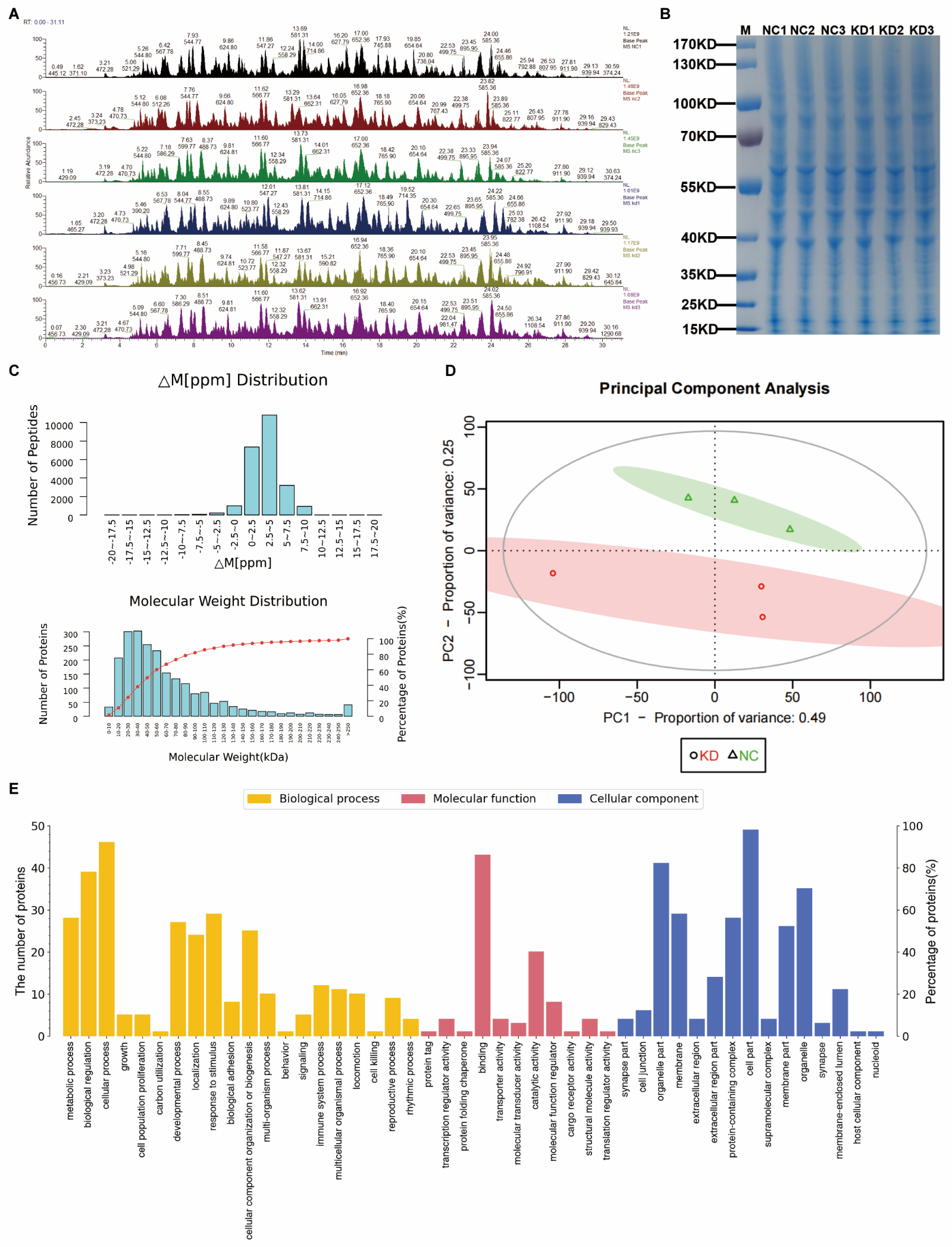
**

**Figure S6. Proteomic Profiling. Related to Figure 6.**

(A) The base peak map of the mass spectrometry and (B) the SDS-PAGE electrophoretogram showed that the quality of the protein was promising, the total amount was sufficient, and the parallelism between samples was ideal.

(C) The distribution diagram of peptide mass deviation (above) shows that the mass deviation of ≥ 95.9% peptides is within 8 ppm, indicating that the instrument is in good condition and the data results are reliable. The distribution map of the relative molecular weight of the identified protein (bottom). The abscissa is the relative molecular weight of the identified protein. The histogram in the left ordinate of protein quantity correspondence diagram represents the identified quantity of proteins with the corresponding relative molecular weight. The accumulation curve in the right ordinate corresponding graph represents the cumulative percentage of proteins with no higher than the corresponding relative molecular weight.

(D) Evaluation of sample consistency by principal component analysis.

(E) GO enrichment analysis of differential proteins.

| **PATIENT** | **GENDER** | **AGE** | **CLASSIFICATION** | **PRIMARY/**  **RECURRENT** | **SITES** | **IDH** | **1p/19q** |
| --- | --- | --- | --- | --- | --- | --- | --- |
| 01 | Female | 36 | WHO 3 | Primary | Left frontal lobe | Mutant | - |
| 02 | Male | 45 | WHO 4 | Primary | Corpus callosum | Wildtype | - |
| 03 | Male | 56 | WHO 4 | Primary | Left parietal lobe | Wildtype | - |
| 04 | Male | 43 | WHO 3 | Recurrent | Right temporal lobe | Mutant | - |
| 05 | Male | 63 | WHO 4 | Recurrent | Vermis cerebelli | Wildtype | - |
| 06 | Male | 71 | WHO 4 | Recurrent | Left frontotemporal lobe | Wildtype | - |
| 07 | Female | 37 | WHO 3 | Primary | Light temporal lobe | Mutant | - |
|  |  |  | WHO 4 | Recurrent |  | Wildtype | - |
| 08 | Male | 38 | WHO 3 | Primary | Light temporal lobe | Mutant | Codeleted |
|  |  |  | WHO 3 | Recurrent |  | Mutant | Codeleted |
| 09 | Male | 45 | WHO 4 | Primary | Left parietal lobe | Wildtype | - |
|  |  |  | WHO 4 | Recurrent |  | Wildtype | - |
| 10 | Male | 49 | WHO 3 | Primary | Left parietooccipital lobe | Mutant | Codeleted |
|  |  |  | WHO 3 | Recurrent |  | Mutant | Codeleted |
| 11 | Male | 51 | WHO 4 | Primary | Left occipitotemporal lobe | Wildtype | - |
|  |  |  | WHO 4 | Recurrent |  | Wildtype | - |
| 12 | Female | 53 | WHO 4 | Primary | Left frontal lobe | Wildtype | - |
|  |  |  | WHO 4 | Recurrent |  | Wildtype | - |
| 13 | Female | 64 | WHO 4 | Primary | Left parietal lobe | Wildtype | - |
|  |  |  | WHO 4 | Recurrent |  | Wildtype | - |
| 14 | Male | 66 | WHO 4 | Primary | Left frontotemporal lobe | Wildtype | - |
|  |  |  | WHO 4 | Recurrent |  | Wildtype | - |
| 15 | Male | 71 | WHO 4 | Primary | Right occipital lobe | Wildtype | - |
|  |  |  | WHO 4 | Recurrent |  | Wildtype | - |

**Table S1. Characteristics of the 15 malignant glioma patients included in this study. Samples obtained from patients 01 to 06 were used for scRNA-seq analysis, and the samples obtained from patients 07 to 15 were used for IHC.**

| **Patient** | **Cells(filtered)** | **UMIs** | **UMI/Cells** | **Gene.mean** | **gene.median** | **Reads** | **Reads/Cells** |
| --- | --- | --- | --- | --- | --- | --- | --- |
| 01 | 3367 | 12484203 | 3707.81 | 2176.39 | 1911 | 363092585 | 35257.99 |
| 02 | 2648 | 24016164 | 9069.55 | 3872.39 | 3602 | 415195973 | 62740.50 |
| 03 | 6049 | 39862246 | 6589.89 | 3365.69 | 2969 | 376451300 | 21036.00 |
| 04 | 2213 | 5620651 | 2539.83 | 1247.59 | 1336 | 377380711 | 35409.98 |
| 05 | 3330 | 21013237 | 6310.28 | 3183.63 | 2874 | 363062655 | 36125.98 |
| 06 | 5403 | 25376624 | 4696.77 | 2729.35 | 2409 | 319998994 | 24695.47 |

**Table S2. Characteristics of the 6 malignant glioma samples used for scRNA-seq analysis. UMI: unique molecular identifier, equivalent to a unique detected transcript.**

| **reference gene** | **forward primer** | **reverse primer** |
| --- | --- | --- |
| GAPDH | TGACTTCAACAGCGACACCCA | CACCCTGTTGCTGTAGCCAAA |
| **target gene** | **forward primer** | **reverse primer** |
| HIF1A | TGAAGTGTACCCTAACTAGCCG | TTCACAAATCAGCACCAAGC |

**Table S3. The information of forward and reverse primers used for qPCR.**
